# Multi-Material Additive Manufacturing of Sustainable Innovative Materials and Structures

**DOI:** 10.3390/polym11010062

**Published:** 2019-01-04

**Authors:** Rupinder Singh, Ranvijay Kumar, Ilenia Farina, Francesco Colangelo, Luciano Feo, Fernando Fraternali

**Affiliations:** 1 Department of Production Engineering, Guru Nanak Dev Engineering College, Ludhiana 141006, India; rupindersingh78@yahoo.com (R.S.); ranvijayk12@gmail.com (R.K.); 2 Department of Engineering, University of Naples Parthenope, 80143 Naples, Italy; francesco.colangelo@uniparthenope.it; 3 Department of Civil Engineering, University of Salerno, 84084 Fisciano (SA), Italy; l.feo@unisa.it

**Keywords:** multi-material printing, fused deposition modelling, tensile properties, thermal properties, mechanical meta-materials

## Abstract

This paper highlights the multi-material additive manufacturing (AM) route for manufacturing of innovative materials and structures. Three different recycled thermoplastics, namely acrylonitrile butadiene styrene (ABS), polylactic acid (PLA), and high impact polystyrene (HIPS) (with different Young’s modulus, glass transition temperature, rheological properties), have been selected (as a case study) for multi-material AM. The functional prototypes have been printed on fused deposition modelling (FDM) setup as tensile specimens (as per ASTM D638 type-IV standard) with different combinations of top, middle, and bottom layers (of ABS/PLA/HIPS), at different printing speed and infill percentage density. The specimens were subjected to thermal (glass transition temperature and heat capacity) and mechanical testing (peak load, peak strength, peak elongation, percentage elongation at peak, and Young’s modulus) to ascertain their suitability in load-bearing structures, and the fabrication of functional prototypes of mechanical meta-materials. The results have been supported by photomicrographs to observe the microstructure of the analyzed multi-materials.

## 1. Introduction

Today, additive manufacturing (AM) has become one of the most common techniques for fabricating periodic lattices and innovative materials [1,2]. Commercially, many fabrication methods are available, with variable resolutions, including: polyjet 3-D printing; fused deposition modelling (FDM), selective laser sintering (SLS); electron beam melting (EBM); laser lithography; and projection microstereolithography etc. [3,4,5,6,7,8,9,10,11].

FDM is one of the low-cost techniques of AM which is used to prepare the functional prototypes of polymers/composites [12,13,14,15,16,17]. In FDM, parts are built layer by layer by heating a thermoplastic filament to a semi-liquid state and extruding it through a small nozzle per 3D CAD models in STL format [18,19]. The filament is usually 1.75 mm to 3.0 mm [20].

The reported literature highlights that the next generation structures using existing materials via AM will surely need to revolve around cost reduction, improved performance, and advanced structural design [21]. The study conducted for 3D printing of multilateral components of ABS and thermoplastic polyurethane (TPU) reveals, with support of 3D imaging, that interface properties are found in control with good layer connectivity [22]. Multi-material 3D printing potential is going to be a milestone in rapid manufacturing, customized design, and structural applications. Being compatible with functionally graded materials in a single structural form, multi-material 3D printing can potentially be applied in structural engineering applications to get the benefit of combined/hybridized material properties. Multi-material printing provides fast and robust structures with the functionality of all the combined materials [23,24]. It was suggested that the hybrid manufacturing (additive+ subtractive) process can fulfill the demand of high dimensional accuracy, less post-processing, and improved surface properties [25]. Multi-material 3D printing is applicable to new smart 4D structures which can provide specific shape/properties/functionalities [26]. It was highlighted that existing AM techniques, such as FDM, can be modified to hybrid deposition manufacturing (HDM) with embedded components to produce more complex, integrated multi-material components than with traditional techniques [27]. It was reported that build orientation, fabrication parameters, and associated variables can largely affect the connection between the multilateral interfaces during 3D printing [28,29], so these should be optimized to get better mechanical, thermal, and surface properties [30,31].

ABS is a common thermoplastic which is amorphous in nature and has high impact resistance, heat resistance, toughness, and low thermal conductivity with potential application in civil engineering. Generally, two types of ABS are classified: one as ABS for molding and another as ABS for extrusion/printing [32,33]. PLA exhibits a range of crystallinity and mechanical properties between polystyrene and polyethylene terephthalate. The bio-degradability and bio-compatibility are the key advantages of PLA to promote its use in the structural and bio-medical applications [34,35]. ‘HIPS’ is a low-cost polymer which provides ease of fabrication and machining. HIPS has low tensile strength, high impact strength (useful for structural application) when it is required to have low cost impact strength, machinability, and fabrication. It is commercially used in pre-production of prototypes because of its high dimensional stability and ease of fabrication, painting, and joining.

The reported literature reveals that many studies have been performed in the recent past to enhance the properties of thermoplastics by reinforcing them with metals/non-metals through extrusion and finally 3D printing by FDM [12,13,18,19]. But, hitherto, very little has been reported on multi-material printing of thermoplastics to enhance its mechanical properties. Since FDM is one of the cost-effective techniques for printing multi-material components, effort has been made to explore the effect of multi-material printing through FDM for preparation of functional prototypes which may be directly installed for structural applications (e.g., as a light load-bearing element) or next-generation mechanical meta-materials. This paper is an extension of work reported by Singh et al. [20], in which break properties were explored to understand the mechanism of failure in the case of multi-material components. In the present study, peak load properties supported by photomicrographs have been explored to understand the multi-material distribution behavior (in form of multi layers) in the3D printing of functional prototypes. In the present study, ABS, PLA, and HIPS were 3D-printed in multi-nozzle FDM to explore the applicability of the final products in structural applications.

## 2. Materials and Methods

ABS has high toughness, a high degree of moldability, and low thermal conductivity. PLA has good biodegradability/crystallinity. HIPS is low-cost with high impact resistance. These three recycled thermoplastics have been selected for the fabrication/multi-material printing operation with FDM. Table 1 shows the mechanical, thermal, and rheological properties of the feedstock materials (average and standard deviation values for three sets of observations). It should be noted that ABS, PLA, and HIPS have significant differences in their MFI, glass transition temperature, peak load, peak strength, peak elongation, Young’s modulus, and yield stress. The aim of the present study is to fabricate the new part with three combined polymeric layers so that the final product possesses the advantages of all the polymers.

## 3. Experimentation

The experimentation stage consisted of the evaluation of melting and solidification characteristics, glass transition temperature determination, extrusion, and multi-material 3D printing.

### 3.1. Differential Scanning Calorimetry (DSC)

DSC is analytical tool for determination of thermal properties, including melting points, glass transition temperature, solidification temperature, degree of crystallinity, heat capacity rate, etc. These properties are defined under controlled continuous heating (endothermic reaction) and controlled continuous cooling (exothermic reaction). The endothermic reaction was carried at the heating rate of +10 °C/min from 30 °C to 250 °C, whereas the exothermic reaction was carried at −10 °C/min from 250 °C to 30 °C. 

### 3.2. Extrusion by Twin Screw Extrusion (TSE)

In the present case, extrusion with TSE was performed at 230 °C with a screw speed of 50 rpm and an applied load of 10 kg to prepare the feedstock filaments. The extrusion parameters were fixed based on pilot experimentation. The TSE used in the present study can produce 1.75 ± 0.05 mm diameter feedstock filaments with yield of 2–3 m/min under 50 rpm screw speed.

### 3.3. 3D Printing

Commercial open-source FDM setup (Make: Divide by Zero, Model 250i, Mumbai, India) configured with two nozzle heads was used for multi-material 3D printing. The static parameters for the fabrication of the combined parts were:

(i) Diameter of nozzle: Φ0.3 mm
(ii) Diameter of filament: Φ1.75 ± 0.05 mm
(iii) Height of layer: 0.27 mm
(iv) Default printing layers on the outer periphery: 3 + 3 (by adjusting 3 top and 3 bottom layers)
(v) Fill pattern: Rectilinear
(vi) Perimeter speed: 30 mm/sec
(vii) Travel speed: 130 mm/sec
(viii) Extrusion temperature: 250 °C
(ix) Print bed temperature: 55 °C

In the present case study three input parameters were varied for printing (see Table 2):

(i) Infill percentage: 60, 80 and 100%
(ii) Speed of printing: 50, 60 and 70 mm/sec.
(iii) Printing material configuration

The multi-material printing was performed with a total of 12 layers (4 layers of each material, i.e., ABS, PLA, and HIPS). The multi-material printing configurations named as APH, PHA, and HAP mean:

APH: bottom 4 layers of ABS, middle 4 layers of PLA, and top 4 layers of HIPS
PHA: bottom 4 layers of PLA, middle 4 layers of HIPS, and top 4 layers of ABS
HAP: bottom 4 layers of HIPS, middle 4 layers of ABS, and top 4 layers of PLA

**Figure 1:**
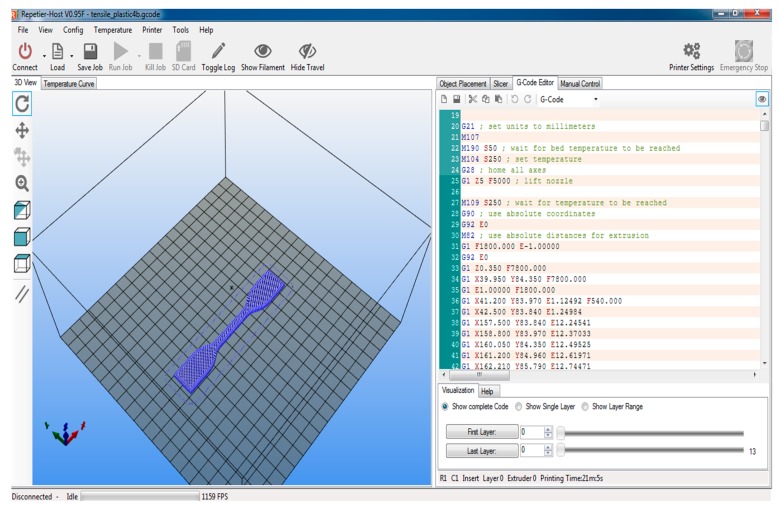
3D benchmark of samples on the printing interface.

Figure 1 shows the 3D view of the benchmark/sample on the printing interface. 

**Figure 2:**
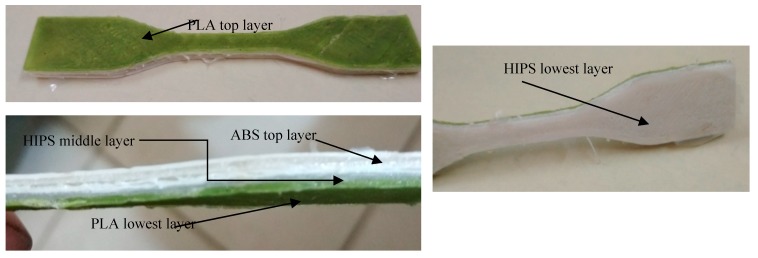
3D-printed multi-material component of ASTM 638 type IV.

Based upon Table 2, 9 specimens (with three repetitions on each setting) of multi-material components were printed (as per Taguchi L9 orthogonal on commercial FDM setup as per ASTM D 638 type IV). The samples composed of single materials (ABS, PLA, and HIPS) were also printed with fixed parametric settings of FDM to analyze the changes in the mechanical strength and the interconnectivity of layers. Figure 2 shows the 3D-printed parts with multi-material layers.

## 4. Results and Discussion

**Figure 3:**
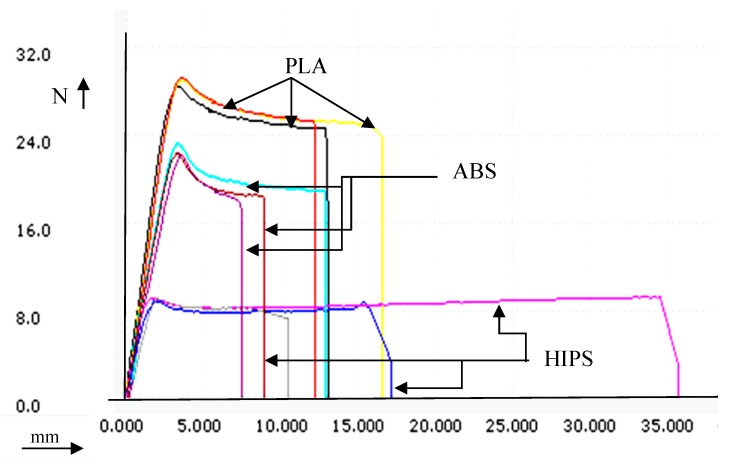
Load vs. deflection curve for extruded feedstock filaments.

It was observed that the extruded feedstock of recycled ABS, PLA, and HIPS resulted in significant differences in mechanical properties. The experimental observations (average of three repeated trails) outlined that, as virgin material, ABS had the greatest Young’s modulus, PLA had the greatest peak load, peak strength, peak elongation, and lowest Young’s modulus and yield stress, whereas HIPS had the lowest peak load, peak elongation, peak strength, and greatest yield stress (See Table 1). Figure 3 shows the load vs. deflection curves of ABS, PLA, and HIPS materials under tensile failure. 

As observed from Figure 3, the selected grade of PLA thermoplastic has the greatest peak load value (see Table 1), followed by ABS and HIPS. Hence, in multi-material structures, if PLA is selected for the outermost layer, followed by ABS (middle layer) and HIPS in the innermost section (especially in arch structures), this will lead to better stability from a load-bearing view point. Similar observations have been made by other investigators [36,37,38].

### 4.1. Thermal Properties

**Figure 4:**
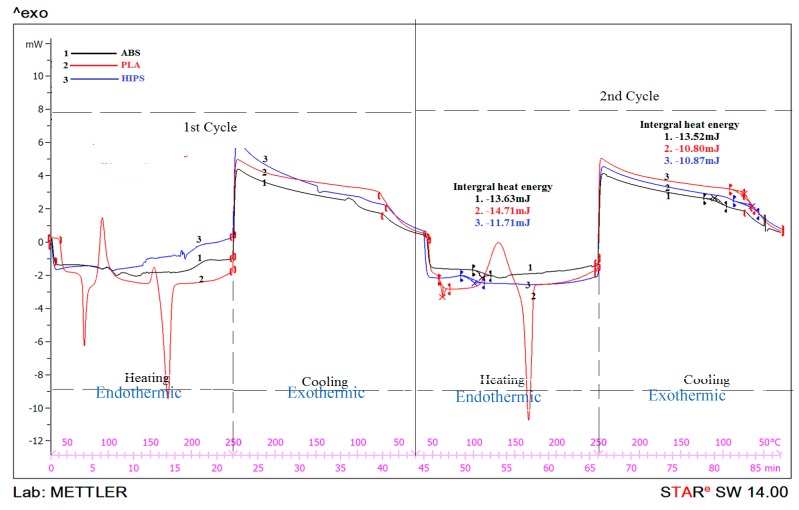
DSC curves for ABS, PLA, and HIPS.

Figure 4 shows the DSC thermographs for ABS, PLA, and HIPS polymers. As observed from Figure 4, ABS, PLA, and HIPS are compatible with each other and have similar ranges of heat integral value. It has been observed that the integral heat input during heating of ABS, PLA, and HIPS was 13.63 mJ, 14.71 mJ, and 11.71 mJ, respectively. Thus, multi-material printing (with proposed combination) may result in better layer connectivity. On the other hand, during solidification of the material, it was observed that ABS, PLA, and HIPS released 13.52 mJ, 10.80 mJ, and 10.87 mJ (which are also in similar range).

As shown in Figure 4, two heating and two cooling cycles were repeated and similar trends of the endothermic and exothermic reactions were observed. Hence, it is ascertained that under repetitive thermal shock, material integrity is not compromised (within the set temperature range). These results are also in line with the observations made otherwise [20]. 

### 4.2. Tensile Properties

The material was tested as per ASTM D 638 type IV (for 12 successive printed layers of ABS/HIPS/PLA) on a tensile testing machine. After the fracture of each sample, data were recorded (see Table 3). Three repetitions were made for each sample setting in order to reduce the experimental error. It was observed that in experiment no. 3 with the APH multi-material configuration, 100% infill percentage and 70 mm/sec printing speed resulted in the greatest peak load, peak strength, and elongation properties and the lowest Young’s modulus, whereas in experiment no. 1 with APH, the 60% infill and 50 mm/sec printing speed configuration resulted in the lowest values of peak load, peak strength, and peak elongation properties. The component/prototype printed in experiment no. 4 had the greatest Young’s modulus. The most important fact was observed in the case of the Young’s modulus for experiments 2, 4, and 9, which resulted in values greater than those of any of the parent materials. Again, the yield stress in experiments 3, 8, and 9 resulted in the values below those of the parent materials. 

**Figure 5:**
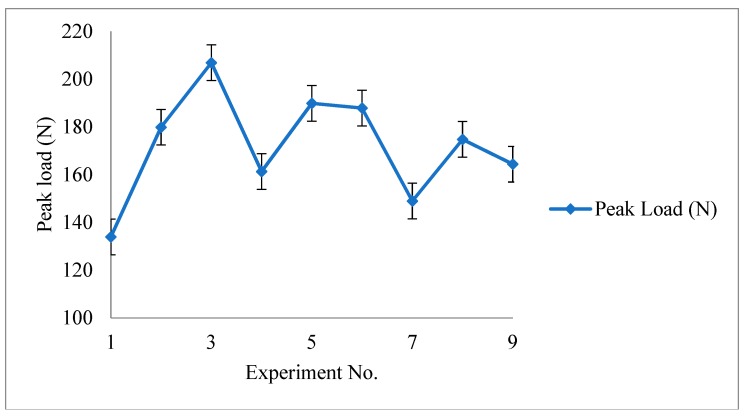
Peak load vs. experiment number (for calculation of standard error).

Based upon Table 3, Figure 5 shows a graphical representation of peak load vs. experiment number (error bars with standard error), which is well in 5% range. Similar results have been attained for all other mechanical properties. 

**Figure 6:**
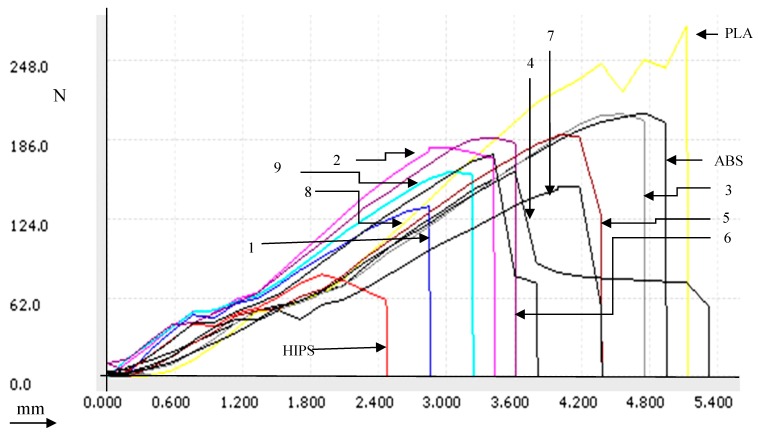
Load vs. deflection curve for tensile fractured components.

**Figure 7:**
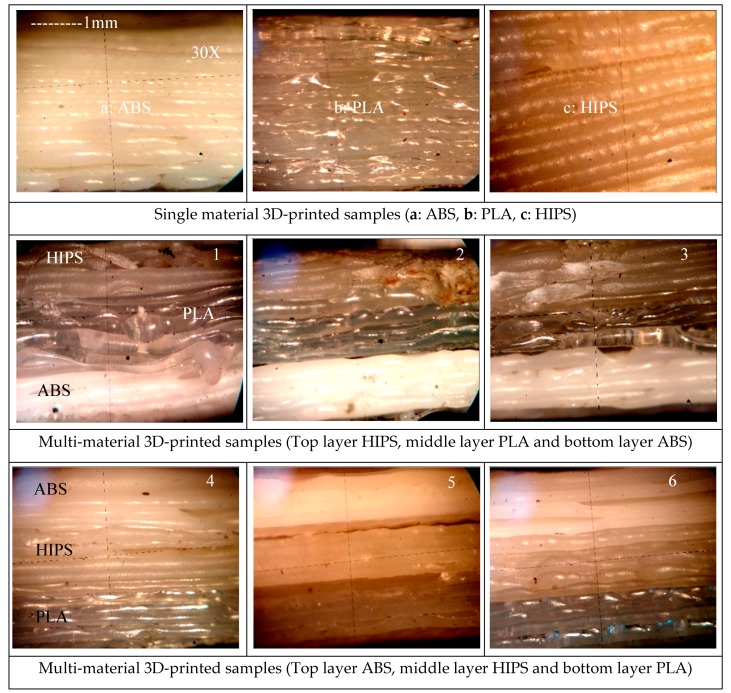

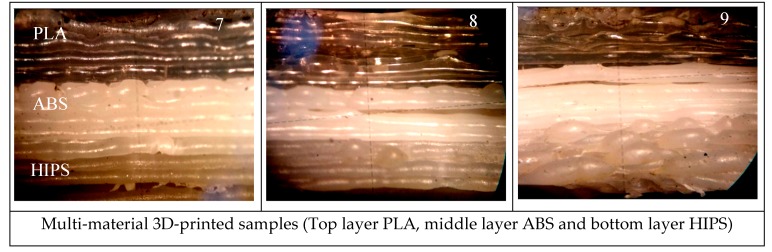
Micrographic observations at 30× magnification with a Tool maker microscope.

3D-printed parts with high part density must have high peak load and low strain values [36,37,38]. As observed from experiments 1–3 (Table 3) with material combination APH, the peak load values follow this behavior, but the peak elongation value at high density is greater, which is contrary to the general behavior. Also, the Young’s modulus value in experiment 2 is higher compared to experiments 1 and 3. This may be because of the fact that the multi-material printed functional prototype has compromised properties, i.e.in tensile loading conditions, the fusion pattern of one material layer on another material layer may have contributed to deviation in the physical-mechanical properties (which is dependent upon many input parameters, including printing speed, rheological properties, material combination, etc.). Similarly, comparing experiments 4–6, better Young’s modulus was observed in experiment 4, whereas, while comparing experiments 7–9, better Young’s modulus was observed in experiment 9. Further, based upon Table 3, Figure 6 shows the load vs. deflection curve for virgin/single printed material as well as multi-material functional prototypes. For better understanding of fused layer deposition, based upon Table 3, photomicrographs were observed with the help of a Mitutoyo Tool maker’s microscope at 30× magnification (see Figure 7). As observed from Figure 7, the single-material printed geometry of ABS, PLA, and HIPS prototypes showed uniform layer orientation, tightly stacked layers, whereas in the case of multi-material prototypes, the uniformity of the layers was compromised. It should be noted that the greatest values of peak load, peak elongation, and peak strength measured by pull out test were achieved in experiment 6 (PHA, 100% infill percentage, and 50 mm/sec printing speed). From photomicrographs of the part printed in experiment 6, it is clear that the layers are tightly stacked and uniformity is maintained (similar to single-material). In the case of experiment 1 where peak load and peak strength had worse values than each single/parent material, the layers were not uniformly packed (See Figure 7).

### 4.3. Pull-Out Test

Pull-out testing is one of the most important considerations for structural applications. The pull-out test was conducted (using the material combinations in Table 3) on all the samples to evaluate the peak load, peak strength, peak elongation, and percentage changes of peak elongation. It was observed that samples 3 and 6 resulted in values of peak load, peak strength greater than HIPS but significantly lower than ABS and PLA. In experiment6, the value of peak elongation resulted in values greater than all the single/parent materials (see Table 4).

**Figure 8:**
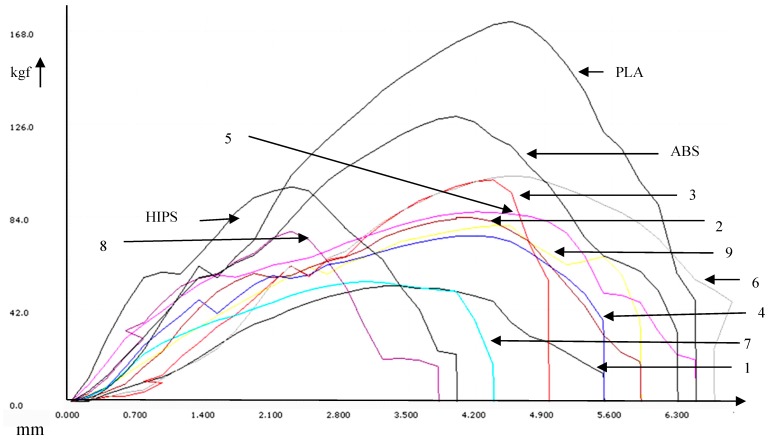
Load vs. deflection curves for multi-material components.

Based upon Table 4, Figure 8 shows the load vs. deflection curves for of the 3D-printed multi-material components. 

## 5. Concluding Remarks

The conclusions from the present study are as follows:

Multi-material 3D printing of recycled ABS, PLA, and HIPS polymers is feasible because these thermoplastics possess similar heat capacities (13.63 mJ for ABS, 14.71 mJ for PLA, and 11.71 mJ for HIPS). 

Tensile properties investigation revealed that the peak strength of HIPS (4.21 MPa) was the lowest of the materials. However, 3D printing of multi-materials resulted in a significant improvement of the tensile strength (10.78 MPa) under controlled input conditions. 

It was observed from the pull-out test that the peak strength of HIPS (27.4 kg/mm^2^) was the lowest of the materials, but multi-material 3D printing of HIPS with ABS and PLA increased its value to 28.81 kg/mm^2^ (at best settings).

Overall, it can be concluded that multi-material printing of various thermoplastics is feasible for functional prototypes and can lead to improvement of their mechanical properties. In light of the structural applications of multi-materials, the future scope lies in the selection of various materials comprising the inner layer (with better compression properties), neutral layers (with moderate compression and tensile properties), and outermost layers (with better tensile properties) selected as per tailor-made requirements. In other words, the limited mechanical properties of some thermoplastics can be used as an advantage in multi-material functional prototypes, e.g., in mechanical meta-materials combining soft and hard modes [8,20]. Moreover, such materials can be also employed to print recycled reinforcing elements to be embedded in an epoxy resin matrix [39] or in a mortar or concrete matrix in order to realize sustainable composite materials [40,41,42,43,44,45,46,47,48,49,50].

With advancements in smart materials for 4D printing and self-assembly applications, multi-material 3D printing can overcome the shortcomings of single materials. Compared to single-material 3D printing, multi-material 3D printing gives more flexibility to functional prototypes (with totally different/enhanced multi-dimensional properties), which basically reduces the required mass of the component and hence the material requirement under different loading conditions. This will help to print more complicated components and to reduce waste.

## Figures and Tables

**Table 1:** Properties of ABS, PLA, and HIPS.

Polymers	HIPS			ABS			PLA		
	OV	SD	SE$\bar{x}$	OV	SD	SE$\bar{x}$	OV	SD	SE$\bar{x}$
MFI (g/10 min)	7.5 ± 0.20	0.16	0.11	8.76 ± 0.16	0.13	0.09	13.52 ± 0.11	0.09	0.06
Young’s modulus (MPa)	112.5 ± 0.12	0.09	0.06	175 ± 0.11	0.09	0.06	47.9 ± 0.10	0.08	0.05
Yield stress (MPa)	3.44 ± 0.21	0.17	0.12	0.49 ± 0.21	0.17	0.12	0.27 ± 0.16	0.13	0.09
Glass transition temp (°C)	100.41 ± 0.16	0.13	0.09	109.76 ± 0.2	0.16	0.11	62.57 ± 0.21	0.17	0.12
Peak load (N)	80.8 ± 0.11	0.08	0.06	207 ± 0.2	0.16	0.11	282.4 ± 0.20	0.16	0.11
Peak strength (MPa)	4.21 ± 0.16	0.13	0.09	10.78 ± 0.11	0.09	0.06	14.71 ± 0.16	0.13	0.09
Peak elongation (mm)	1.9 ± 0.20	0.16	0.11	4.75 ± 0.16	0.13	0.09	5.13 ± 0.16	0.13	0.09
Percentage elongation at peak (%)	3.0 ± 0.11	0.09	0.06	6.0 ± 0.15	0.12	0.08	7.0 ± 0.10	0.08	0.05

Note: OV = Observed value, SD = standard deviation, SE$\bar{x}$ = Standard error of mean.

**Table 2:** Input process variables for multi-material 3D printing on FDM.

Parameters	Level 1	Level 2	Level 3
**Material Combination**	APH	PHA	HAP
**Infill percentage (%)**	60	50	100
**Printing Speed (mm/sec)**	50	60	70

**Table 3:** Mechanical properties of 3D-printed multi-material components.

Exp No.	Material Combination	Infill (%)	Printing (mm/sec)	Peak Load (N)			Peak Strength (MPa)			Peak Elongation (mm)			Percentage Elongation at Peak (%)			Young’s Modulus (MPa)			Yield Stress (MPa)		
				OV	SD	SE$\bar{x}$	OV	SD	SE$\bar{x}$	OV	SD	SE$\bar{x}$	OV	SD	SE$\bar{x}$	OV	SD	SE$\bar{x}$	OV	SD	SE$\bar{x}$
1	APH	60	50	133.9 ± 0.16	0.13	0.09	6.97 ± 0.20	0.16	0.11	2.85 ± 0.11	0.08	0.06	4 ± 0.16	0.13	0.09	72.92 ± 0.22	0.18	0.13	2.73 ± 0.21	0.17	0.12
2	APH	50	60	179.9 ± 0.21	0.17	0.12	9.37 ± 0.11	0.08	0.06	2.85 ± 0.22	0.17	0.12	4 ± 0.16	0.13	0.09	264.58 ± 0.21	0.17	0.12	2.68 ± 0.16	0.13	0.09
3	APH	100	70	206.9 ± 0.11	0.08	0.06	10.78 ± 0.12	0.09	0.06	4.37 ± 0.17	0.14	0.10	6 ± 0.17	0.13	0.09	73.29 ± 0.11	0.08	0.06	0.21 ± 0.20	0.16	0.11
4	PHA	60	60	161.3 ± 0.21	0.17	0.12	8.40 ± 0.16	0.13	0.09	3.04 ± 0.20	0.16	0.11	4 ± 0.22	0.17	0.12	325.00 ± 0.12	0.09	0.06	1.00 ± 0.11	0.08	0.06
5	PHA	80	70	189.9 ± 0.20	0.16	0.11	9.89 ± 0.12	0.09	0.06	3.99 ± 0.13	0.09	0.06	5 ± 0.21	0.17	0.12	79.17 ± 0.05	0.04	0.02	4.54 ± 0.2	0.08	0.05
6	PHA	100	50	187.9 ± 0.11	0.08	0.06	9.79 ± 0.20	0.16	0.11	3.23 ± 0.12	0.09	0.06	4 ± 0.1	0.08	0.05	108.33 ± 0.12	0.09	0.06	5.13 ± 0.16	0.13	0.10
7	HAP	60	70	149.0 ± 0.11	0.08	0.06	7.76 ± 0.22	0.17	0.12	3.99 ± 0.16	0.13	0.09	5 ± 0.11	0.08	0.06	85.42 ± 0.18	0.14	0.10	0.28 ± 0.11	0.08	0.06
8	HAP	80	50	174.8 ± 0.16	0.13	0.09	9.10 ± 0.11	0.08	0.06	3.42 ± 0.12	0.08	0.06	5 ± 0.21	0.17	0.12	161.84 ± 0.12	0.08	0.06	0.17 ± 0.21	0.17	0.12
9	HAP	100	60	164.4 ± 0.11	0.08	0.06	8.56 ± 0.16	0.13	0.09	3.61 ± 0.13	0.10	0.07	5 ± 0.16	0.13	0.09	249.67 ± 0.13	0.10	0.07	0.16 ± 0.16	0.13	0.09

**Table 4:** Pull-out properties of 3D-printed components.

Experiment no.	Peak Load (kgf)			Peak Strength (kg/mm^2^)			Peak Elongation (mm)			Percentage Elongation at Peak (%)		
	OV	SD	SE$\bar{x}$	OV	SD	SE$\bar{x}$	OV	SD	SE$\bar{x}$	OV	SD	SE$\bar{x}$
1	52.8 ± 0.15			14.83 ± 0.16	0.13	0.09	3.23 ± 0.10			2 ± 0.15	0.12	0.08
2	80.1 ± 0.16	0.13	0.09	22.49 ± 0.12	0.09	0.06	4.37 ± 0.16	0.13	0.09	2 ± 0.15	0.12	0.08
3	100.7 ± 0.11	0.08	0.06	28.27 ± 0.12	0.09	0.06	4.18 ± 0.12	0.09	0.06	2 ± 0.10	0.08	0.06
4	75.4 ± 0.11	0.08	0.06	21.17 ± 0.21	0.17	0.12	4.18 ± 0.06	0.04	0.03	2 ± 0.20	0.16	0.11
5	86.2 ± 0.11	0.08	0.06	24.2 ± 0.21	0.17	0.12	4.18 ± 0.16	0.13	0.09	2 ± 0.10	0.08	0.06
6	102.6 ± 0.21	0.17	0.12	28.81 ± 0.11	0.08	0.06	4.56 ± 0.11	0.08	0.06	2 ± 0.10	0.08	0.06
7	54.7 ± 0.16	0.13	0.09	15.36 ± 0.17	0.14	0.09	2.85 ± 0.16	0.13	0.09	1 ± 0.20	0.16	0.12
8	83.6 ± 0.11	0.08	0.06	23.47 ± 0.21	0.17	0.12	3.99 ± 0.21	0.17	0.12	2 ± 0.10	0.08	0.06
9	77.4 ± 0.16	0.13	0.09	21.73 ± 0.22	0.18	0.13	2.28 ± 0.16	0.13	0.09	1 ± 0.15	0.12	0.08
**ABS**	129.5 ± 0.20	0.16	0.11	36.36 ± 0.17	0.14	0.09	3.99 ± 0.11	0.08	0.06	2 ± 0.15	0.12	0.08
**PLA**	172.6 ± 0.21	0.17	0.12	48.46 ± 0.22	0.18	0.13	4.37 ± 0.23	0.18	0.13	2 ± 0.20	0.16	0.11
**HIPs**	97.6 ± 0.12	0.09	0.06	27.4 ± 0.11	0.08	0.06	2.28 ± 0.23	0.18	0.13	1 ± 0.20	0.16	0.11
